# Involvement of CRFR_1_ in the Basolateral Amygdala in the Immediate Fear Extinction Deficit

**DOI:** 10.1523/ENEURO.0084-16.2016

**Published:** 2016-11-02

**Authors:** Fiona Hollis, Yannick Sevelinges, Jocelyn Grosse, Olivia Zanoletti, Carmen Sandi

**Affiliations:** Brain Mind Institute, École Polytechnique Fédérale de Lausanne, 1015 Lausanne, Switzerland

**Keywords:** basolateral amygdala, corticotropin-releasing factor, fear conditioning, immediate-extinction deficit

## Abstract

Several animal and clinical studies have highlighted the ineffectiveness of fear extinction sessions delivered shortly after trauma exposure. This phenomenon, termed the immediate extinction deficit, refers to situations in which extinction programs applied shortly after fear conditioning may result in the reduction of fear behaviors (in rodents, frequently measured as freezing responses to the conditioned cue) during extinction training, but failure to consolidate this reduction in the long term. The molecular mechanisms driving this immediate extinction resistance remain unclear. Here we present evidence for the involvement of the corticotropin releasing factor (CRF) system in the basolateral amygdala (BLA) in male Wistar rats. Intra-BLA microinfusion of the CRFR_1_ antagonist NBI30775 enhances extinction recall, whereas administration of the CRF agonist CRF_6–33_ before delayed extinction disrupts recall of extinction. We link the immediate fear extinction deficit with dephosphorylation of GluA1 glutamate receptors at Ser^845^ and enhanced activity of the protein phosphatase calcineurin in the BLA. Their reversal after treatment with the CRFR_1_ antagonist indicates their dependence on CRFR_1_ actions. These findings can have important implications for the improvement of therapeutic approaches to trauma, as well as furthering our understanding of the neurobiological mechanisms underlying fear-related disorders.

## Significance Statement

Trauma-related disorders are costly, highlighting the need to understand the reduction of fear through extinction learning for the development of better therapies. When extinction programs are applied too soon after the traumatic event, numerous studies have found them to be ineffective, although the underlying mechanisms were unclear. Here we confirm that futility of immediate extinction and provide a mechanistic explanation. Using a pharmacological approach, we show evidence for the involvement of the corticotropin releasing factor (CRF) system in the basolateral amygdala in this extinction deficit. We link this involvement with downstream molecular targets of the CRF system that are critical in synaptic plasticity, thus explaining the futility of immediate extinction and providing further insight into fear-related disorders.

## Introduction

Trauma-related disorders impose a high burden on both individuals and society (Kessler et al., 2012), inspiring numerous studies of the mechanisms underlying fear extinction learning (Pape and Pare, 2010; Milad and Quirk, 2012). Reduction of fear responses to previously fearful stimuli through fear extinction learning is a complex process involving a broad network of brain structures, including the basolateral nucleus of the amygdala (BLA; Sotres-Bayon et al., 2004; Quirk and Mueller, 2008, Herry et al., 2010). Several molecular targets have been identified for the development of therapeutic interventions for post-traumatic stress disorder (PTSD) and other fear-related disorders (Milad and Quirk, 2012; Singewald et al., 2015). Among them, emerging evidence points to a key role for the central corticotropin releasing factor (CRF) system, which is well known for its role in the regulation of stress, fear, stressful learning, and anxiety responses (Bale, 2005; Nemeroff et al., 2006; Regev and Baram, 2014). CRF, a 41–amino acid peptide involved in the activation of the hypothalamic-pituitary-adrenal axis, also exerts extrahypothalamic actions in different brain regions through activation of two G-protein–coupled receptors, CRFR_1_ and CRFR_2_. Both the BLA and the central nucleus of the amygdala contain CRF-expressing neurons, and the BLA presents particularly high CRFR_1_ densities (Korosi and Baram, 2008).

Recently, a key role for the CRF system in impaired extinction processes has been suggested. In humans, enhanced CRF levels found in the CSF of PTSD patients have indicated a link between increased CRF concentrations and disrupted fear extinction observed in PTSD and anxiety disorders (Bangasser and Kawasumi, 2015; Gafford and Ressler, 2015). In rats, administration of CRF into the lateral amygdala before fear recall testing in formerly fear-conditioned animals induced enhanced freezing responses to the conditioned stimulus (Isogawa et al., 2013). Additionally, pharmacological enhancement of CRF in the BLA impaired long-term retention of fear extinction, whereas CRFR_1_ antagonism had the opposite effect (Abiri et al., 2014), supporting a detrimental role of CRF in the BLA in the consolidation of cued fear extinction. Furthermore, cell-specific genetic disruption of GABAAα1 within CRF-expressing neurons in mice was found to specifically impair fear extinction (i.e., not affecting fear conditioning or retention) processes, whereas systemic administration of a CRF antagonist partially rescued the fear extinction deficit in these mice (Gafford et al., 2012). Accordingly, an overactive CRF system in the BLA seems to interfere with extinction processes.

Importantly, it is not known whether the CRF system is involved in a particularly challenging extinction case known as “immediate fear extinction deficit” (Maren, 2014). This refers to the ineffectiveness of fear extinction programs, frequently observed both in animals and humans, when extinction training is administered soon (e.g., from minutes to a few hours) after fear conditioning. Specifically, despite exhibiting decreased within-session freezing during extinction training, subjects fail to maintain this response over long-term retention intervals (Maren and Chang, 2006; Schiller et al., 2008; Woods and Bouton, 2008; Chang and Maren 2009; Archbold et al., 2010; but see Myers et al., 2006). Given that, in addition to its involvement in the acquisition and consolidation of fear conditioning (Sah et al., 2008), the BLA has been implicated in fear extinction (Sotres-Bayon et al., 2004; Quirk and Mueller, 2008; Herry et al., 2010), we hypothesized that mechanisms that contribute to fear conditioning in the BLA might underlie the effectiveness of immediate extinction trials. Converging lines of evidence support the involvement of the BLA CRF system in the immediate extinction deficit. First, acute stress has been shown to rapidly induce CRF release in the amygdala (Pich et al., 1995; Merali et al., 1998), leading to activation of CRFR_1_ in the BLA in the consolidation of fear learning (Roozendaal et al., 2002, 2008; Hubbard et al., 2007). Second, CRF increases excitability of CRFR_1_-containing BLA projection neurons (Rainnie et al., 1992) and induces long-lasting increases in the amplitude of field postsynaptic potentials in the BLA (Sandi et al., 2008; Ugolini et al., 2008). These effects are reversed by antagonizing CRFR_1_, but not CRFR_2_ (Rainnie et al., 1992; Ugolini et al., 2008). Finally, whereas CRF infusions in the lateral amygdala before long-term memory testing enhanced freezing responses to the conditioned stimulus (Isogawa et al., 2013), CRFR_1_ antagonism in the BLA facilitated long-term retention of fear extinction (Abiri et al., 2014). Therefore, we evaluated the involvement of the CRF system in the amygdala in the minimal fear suppression induced by extinction training given shortly after fear conditioning and explored the molecular machinery involved.

## Materials and Methods

### Subjects

Male Wistar rats (Charles River, L’Arbresle, France) weighing 250–300 g at the start of the experimentation served as subjects and were singly housed in polypropylene cages (34 × 29 × 17 cm) lined with abundant pine shavings. Animals had *ad libitum* access to food and water and were maintained in constant temperature (23°C) and lighting (0700–1900) conditions. All experiments were performed during the light phase. Animals were allowed to habituate to the vivarium for 1 week and were then handled for 2 min on 3 days before the beginning of all experiments.

All procedures were conducted in conformity with the École Polytechnique Fédérale de Lausanne’s guidelines for animal experimentation. All efforts were made to minimize suffering and reduce the number of animals used.

### Elevated plus maze

Before experiments, anxiety-related behavior was measured using the Elevated Plus Maze (EPM) according to the procedure described in Herrero et al. (2006). As previous reports indicate that CRF antagonist NBI30775 affects subjects differently depending on their natural anxiety level (Sandi et al., 2008), all groups were matched according to similar scores in this test. EPM sessions for all experiments were conducted 4–7 d before the first fear conditioning session.

### Fear conditioning

#### Conditioning session

The training cage (Context A) consisted of a Plexiglas transparent chamber (30 × 37 × 25 cm; Panlab, Barcelona, Spain) that was positioned inside a sound-attenuating chamber. This chamber was constructed of black stainless steel walls of smooth texture, with a ceiling and door made of Plexiglas. The floor consisted of 20 steel rods wired to a shock source and solid-state scrambler for the delivery of foot shocks. Conditioning took place in a single session. After 3 min of free exploration, rats received five pairings of a 2-s conditioned-stimulus (CS) tone (80 dB, 2000 Hz) and a 0.5-s unconditioned-stimulus foot shock (0.6 mA). The intershock interval was 60 s. Subjects were removed from the chambers 58 s after the final shock presentation (thus, the training session lasted 8 min) and left undisturbed in their home cage until the extinction session.

#### Extinction session

Extinction of cued fear learning took place in a different context (Context B). The context shape was modified, the grid was replaced by a plastic smooth floor, and visual and odor cues were changed. Animals were free to explore the environment during the first 3 min, and then 70 CS were presented every 40 s. Depending on the protocol, the extinction session took place 30 min, 3 h, or 24 h after training. For each behavioral experiment, separate groups of animals were placed in the extinction context without any CS presentation as controls.

#### Testing session

Forty-eight hours after training, extinction memory was assessed in Context B. After 3 min of free exploration, the rats received five CS presentations with an intertrial interval of 60 s. Rats were removed from the chambers 58 s after the final CS presentation (8 min total duration)

In all sessions, behavior was monitored with a camera connected to a videorecorder for offline analysis, which was performed by an experimenter blind to the animal’s experimental condition. Fear was assessed by measuring the percentage of time spent freezing, characterized by a crouching posture and an absence of any visible movement except breathing.

### Surgery and amygdala microinfusions

Animals were anaesthetized with i.p. ketamine (70 mg/kg) and xylazine (6 mg/kg). They were then implanted with two 18-mm stainless steel guide cannulae (23-gauge; Plastic One, Roanoke, VA) using a standard stereotaxic frame (Kopf Instruments, Bioseb, France). Cannulae were bilaterally implanted at the BLA coordinates (anteroposterior, –2.8 mm relative to bregma; lateral, ±5.1 mm from midline; ventral, –5.5 mm from dura). The tips of the cannulae were aimed 2 mm above the intended area. The cannulae were fixed to the skull with dental acrylic cement. Stylets were inserted into the guide cannulae to prevent clogging. Rats were given 1 week to recover from surgery, after which they received the EPM and fear sessions as described above.

All animals were handled individually for approximately 1–2 min each day during the 2–3 days preceding infusion to habituate them to the infusion procedure. Immediately after training, rats were gently restrained, stylets were removed, and injection needles (30 gauges) were inserted, extending 2 mm from the tip of the guide cannula. The injection needles were connected via polyethylene tubing to two 10-μl Hamilton microsyringes driven by an automated microinfusion pump (Harvard Apparatus). The needles were then left in position for an additional minute to enable diffusion of the solution into the tissue and minimize dragging of the liquid along the injection track.

### Drugs

NBI30775 (3-[6-(dimethylamino)-4-methyl-pyrid-3-yl]-2,5-dimethyl-*N*,*N*-dipropyl-pyrazolo[2,3-a]pyrimidin-7-amine), a nonpeptide CRFR_1_ antagonist (also known as R121919) was a gift from Dimitri Grigoriadis (Neurocrine Inc., San Diego, CA). It was dissolved in dimethylsulfoxide (DMSO) and bilaterally infused in the BLA at different concentrations (0.1, 0.3, 1, or 10 µg in 0.5 µl) at a rate of 0.3 µl/min immediately after fear conditioning. Vehicle infusions of DMSO were administered in a similar manner. The infusion volume for NBI30775 experiments was 0.5 µl/hemisphere. Depending on the experiment, animals were subject to extinction 30 min thereafter or left undisturbed until the testing session 48 h and 7 d after training.

The CRF agonist CRF_6–33_ (Sigma-Aldrich, St. Louis, MO) was dissolved in saline and infused bilaterally in the BLA at a concentration of 0.1 µg in 0.2 µl, at a rate of 0.3 µl/min just before a delayed (24 h after training) extinction session. The infusion volume for agonist experiments was 0.2 µl/hemisphere. Animals were tested 48 h after training. Vehicle infusions of saline were administered in a similar manner.

After completion of behavioral experiments, animals were overdosed with sodium pentobarbital (100 mg/kg i.p.). The brains were removed and immediately frozen at –50°C in isopentane and stored at –20°C. Coronal sections (40 µm thick) were stained with thionine for histological checking. Of the 84 implanted animals, 12 were discarded due to misplacement of one or both cannulae.

### Western blot

#### Tissue preparation

Immediately after decapitation, the amygdala was rapidly dissected out and frozen at –80°C until processing. Tissue was homogenized in 10 volumes of ice-cold sucrose (0.32 m) and HEPES (5 mm) buffer that contained a cocktail of protease inhibitors (Complete TM, Roche, West Sussex, UK) with 16 strokes and centrifuged at 1000 × *g* for 5 min. The resulting total fraction pellet was resuspended in Krebs buffer with 1% NP40, incubated at 4°C for 40 min, and centrifuged at 10,000 × *g* for 20 min at 4°C. Protein concentration for each sample was estimated by bicinchoninic acid protein analysis (Bio-Rad, Hercules, CA).

#### Quantification of phosphorylation of the AMPA GluA1 subunits

Ten micrograms of protein were loaded in each well and separated on 10% (w/v) SDS-PAGE and transferred (70V, 90 min) to a nitrocellulose membrane (Whatman, Maidstone, UK). Membranes were incubated overnight at 4°C with a rabbit anti–phospho-Ser^845^ GluA1 polyclonal antibody (detects phosphorylation on GluA1 serine-845 only, 1:5000, cat. P1160-845, RRID: AB_2492128; PhosphoSolutions, Aurora, CO), a rabbit anti–phospho-ser^831^ GluA1 monoclonal antibody (detects phosphorylation on GluA1 serine-831 only, 1:5000, cat. 04-823, RRID: AB_1977218; Merck-Millipore, Billerica, MA), and a rabbit anti-GluA1 polyclonal antibody (detects GluA1 irrespective of modifications, 1:10,000, cat. ADI-905-416-1, RRID: AB_2039139; Assay Designs). Monoclonal mouse anti-actin (1:5000, cat. A3853, RRID: AB_262137; Sigma-Aldrich), and a mouse monoclonal anti-GAPDH (1:40,000, cat. Ab8245, RRID: AB_2107448; Abcam, Cambridge, UK) were incubated as loading controls. The blots were washed with PBS plus Tween, incubated for 1 h with a secondary antibody, a goat anti-rabbit IgG horseradish peroxidase conjugate (1:5000, cat. G-21234, RRID: AB_1500696; Thermo Fisher Scientific, Waltham, MA), and a goat anti-mouse IgG peroxidase conjugate for loading controls (1:5000, cat. 401215, RRID: AB_10682749; Calbiochem) and developed using an enhanced chemiluminescence system (Pierce, Rockford, IL). Bands were revealed with a ChemiDoc imaging system (Bio-Rad) for optimum exposure time. Images were analyzed using QuantityOne software v4.6.3 (Bio-Rad), where the adjusted volume was calculated for each band. For each group, the value of pGluA1 Serine subunit was normalized to total GluA1 after normalization to loading controls. To assess changes relative to the basal state, each experimental group is reported as a percentage of home cage group values.

### Calcineurin activity ELISA assay

Immediately after rapid decapitation, brains were dissected out and flash-frozen in ice-cold isopentane. The basolateral amygdala was tissue punched on a freezing cryostat and stored at –80°C for further processing. Samples were homogenized and processed according to the manufacturer’s protocol for the Calcineurin Cellular Activity kit (BML-AK816; Enzo Life Sciences, Lausen, Switzerland), with the following adjustments. Punches were homogenized in 200 µl lysis buffer with protease inhibitor using a motorized pestle and passed through a desalting column to remove excess phosphate. Calcineurin activity was then measured according to manufacturer’s specifications.

### Data analyses

Intergroup comparisons were evaluated using Student’s unpaired *t*-test and one- or two-way ANOVA followed by the Fisher test for post hoc analysis where appropriate. Differences were considered significant if *p* < 0.05. Superscript letters listed with *p*-values correspond to the statistical tests shown in Table 1. For Western blot data and calcineurin activity, data are shown as percentage of home cage controls.

**Table 1:** Summary of statistics for all experiments.

**Line**	**Figure**	**Description**	**Data structure**	**Type of test**	**Power**
a	1*B*	EPM: time spent on the open arms	Normal distribution	2-way ANOVA	1
b	1*C*	Extinction training: effect of trial	Normal distribution	3-way repeated ANOVA	1
c	1*C*	Extinction training: extinction × trial interaction	Normal distribution	3-way repeated ANOVA	0.95
d	1*C*	Extinction training: interval × trial interaction	Normal distribution	3-way repeated ANOVA	0.45
e	1*C*	Extinction training: extinction interval × trial	Normal distribution	3-way repeated ANOVA	0.12
f	1*D*	Extinction test: effect of extinction	Normal distribution	2-way ANOVA	0.99
g	1*D*	Extinction test: effect of interval	Normal distribution	2-way ANOVA	0.94
h	1*D*	Extinction test: extinction × interval interaction	Normal distribution	2-way ANOVA	0.69
I	1*D*	Extinction test: freezing (30-min EXT vs. 30-min No-EXT)	Normal distribution	Fisher’s post hoc test	0.24
J	1*D*	Extinction test: freezing (3-h EXT vs. 3-h No-EXT)	Normal distribution	Fisher’s post hoc test	1
k	1*D*	Extinction test: freezing (24-h EXT vs. 24-h No-EXT)	Normal distribution	Fisher’s post hoc test	1
l	1*D*	Extinction test: freezing (3-h EXT vs. 24-h EXT)	Normal distribution	Fisher’s post hoc test	1
m	1*E*	Correlation: 30-min postconditioning train vs. test	Normal distribution	Linear correlation	*R* ^2^ = 0.74
n	1*F*	Correlation: 24-h postconditioning train vs. test	Normal distribution	Linear correlation	*R* ^2^ = 0.52
o	Not shown	Correlation: 3-h postconditioning train vs. test	Normal distribution	Linear correlation	*R* ^2^ = 0.28
p	2*B*	EPM: time spent on the open arms	Normal distribution	1-way ANOVA	1
q	2*D*	Extinction training: effect of trial	Normal distribution	2-way repeated ANOVA	1
r	2*E*	Extinction test: effect of drug	Normal distribution	1-way ANOVA	0.81
s	2*E*	Extinction test: freezing (VEH vs. 10 µg)	Normal distribution	Fisher’s post hoc test	1
t	2*F*	Extinction test, 7 d: effect of drug	Normal distribution	1-way ANOVA	0.81
u	2*F*	Extinction test, 7 d: freezing (VEH vs. 1 µg)	Normal distribution	Fisher’s post hoc test	1
v	2*F*	Extinction test-7d: freezing (VEH vs. 10 µg)	Normal distribution	Fisher’s post hoc test	1
w	2*G*	Consolidation control: freezing (VEH vs. 10 µg)	Normal distribution	Student’s 2-tailed t-test	0.96
x	3*B*	EPM: time spent on the open arms	Normal distribution	Student’s 2-tailed t-test	0.8
y	3*C*	Extinction training: effect of trial	Normal distribution	2-way repeated ANOVA	0.91
z	3*D*	Extinction test: effect	Normal distribution	Student’s 2-tailed t-test	0.83
aa	4*B*	EPM: time spent on the open arms	Normal distribution	2-way ANOVA	1
bb	4*C*	Protein: extinction training effect of trial	Normal distribution	2-way repeated ANOVA	1
cc	4*C*	Protein: extinction training effect of interval	Normal distribution	2-way repeated ANOVA	0.3
dd	4*C*	Protein: extinction training: effect of group	Normal distribution	2-way repeated ANOVA	0.44
ee	4*C*	Protein: extinction training: interaction	Normal distribution	2-way repeated ANOVA	0.29
ff	4*D*	Protein: 30-min interval	Normal distribution	1-way ANOVA	0.79
gg	4*D*	Protein: 30-min (HOME vs. CtxB no CS)	Normal distribution	Fisher’s post hoc test	0.12
hh	4*D*	Protein: 3-min (HOME vs. CtxB CS)	Normal distribution	Fisher’s post hoc test	1
ii	4*D*	Protein: 30-min (HOME vs. CtxB-3-min CS	Normal distribution	Fisher’s post hoc test	1
jj	5*B*	EPM: time spent on the open arms	Normal distribution	1-way ANOVA	1
kk	5*C*	Protein: Extinction training: effect of “trial”	Normal distribution	2-way repeated ANOVA	1
ll	5*C*	Protein: extinction training: effect of treatment	Normal distribution	2-way repeated ANOVA	0.7
mm	5*C*	Protein: extinction training: interaction	Normal distribution	2-way repeated ANOVA	0.47
nn	5*E*	Protein (VEH vs. HOME)	Normal distribution	Student’s 2-tailed t-test	1
oo	5*E*	Protein (NBI vs. HOME)	Normal distribution	Student’s 2-tailed t-test	0.06
pp	5*G*	Protein: effect of treatment	Normal distribution	2-way ANOVA	0.11
qq	6*B*	EPM: time spent on the open arms	Normal distribution	2-way ANOVA	1
rr	6*C*	Calcineurin activity: effect of NBI	Normal distribution	2-way ANOVA	0.79
ss	6*C*	Calcineurin activity (VEH CtxB-CS vs. home cage)	Normal distribution	Fisher’s post hoc test	0.99
tt	6*C*	Calcineurin activity (NBI CtxB-CS vs. home cage)	Normal distribution	Fisher’s post hoc test	0.07

## Results

In all experiments, we verified that freezing levels during fear conditioning did not differ for the different experimental groups included. For the sake of clarity, these analyses are reported in Table 2.

**Table 2.** Summary of statistics for fear conditioning sessions.

**Experiment**	**Group**	**Mean**	**SEM**	**Test**	***p*-value**
Figure 1	No-Ext, 30 min	80.154	3.378	3-way repeated ANOVA	
	Ext, 30 min	77.576	2.659		Time: < 0.0001
	No-Ext, 3 h	100.147	12.016		Time × extinction: 0.51
	Ext, 3 h	91.933	10.933		Time × interval: 0.51
	No-Ext, 24 h	88.575	1.827		Interaction: 0.17
	Ext, 24 h	78.4	2.728		
Figure 2	VEH	81.8	3.872	1-way repeated ANOVA	
	0.1 µg	85.033	2.789		Time: < 0.0001
	0.3 µg	83.571	3.521		Group: 0.21
	1 µg	69.486	8.752		
	10 µg	77.28	3.238		
Figure 3	VEH	75.566	7.053	1-way repeated ANOVA	Time: < 0.0001
	0.1 µg	84.853	3.149	Group: 0.89	

### Immediate extinction sessions result in inefficient extinction

**Figure 1.**
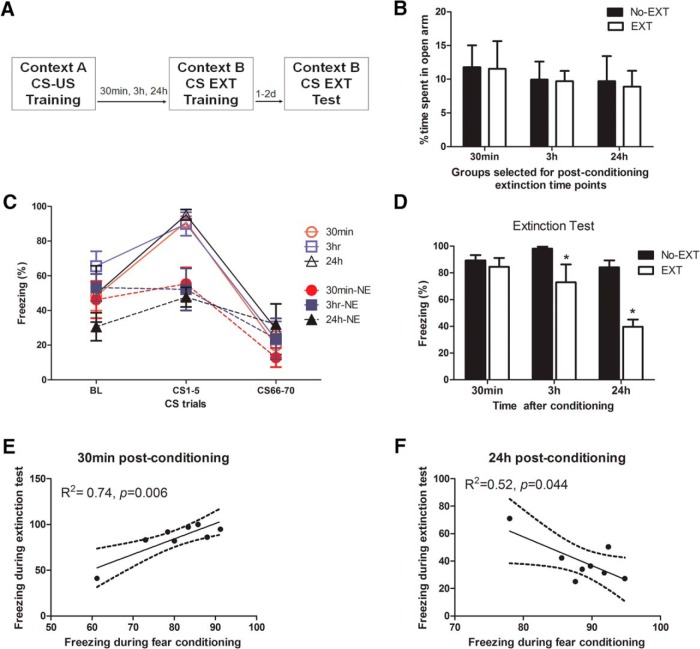
Efficient fear extinction is dependent on the interval between conditioning and extinction sessions. Animals were separated into EXT and No-EXT (NE) groups (***A***) balanced for anxiety-like behavior on the elevated plus maze (***B***) and conditioned to similarly associate a foot shock with a tone assessed by levels of freezing (see also Table 2). Animals in the EXT group were then exposed to an extinction session (30 min, 3 h, or 24 h after conditioning), with all interval groups showing similar patterns of extinction (***C***, open symbols). Animals in the No-EXT group were exposed to the Context B arena without tones, and each subgroup showed similar levels of freezing during this period (***C***, filled symbols). Animals exposed to extinction sessions immediately after conditioning (30 min) exhibited levels of freezing similar to those of the No-EXT group during the extinction test (***D***). Animals exposed to delayed extinction sessions (3 or 24 h) exhibited significantly reduced freezing levels. BL, baseline freezing during the first 3 min pretone. The freezing behavior during the extinction test in animals exposed to an extinction session 30 min after conditioning was positively and significantly correlated with the amount of fear shown during the initial fear training sessions (***E***), whereas those receiving an extinction session 24 h after conditioning had a significant negative correlation between freezing behavior in the extinction test versus fear training (***F***). Data are depicted as mean percentage of time spent freezing ± SEM. *Significant difference (*p* < 0.05) with the corresponding No-EXT group; *n* = 8/group.

To identify appropriate experimental conditions to evaluate mechanisms underlying the immediate fear extinction deficit phenomenon, we first examined the efficiency of performing extinction training at several time points post-conditioning (Fig. 1*A*). Here, efficiency means that we examined whether animals exposed to extinction training at particular post-conditioning intervals were able to demonstrate significantly reduced freezing levels during a test, compared to corresponding non-extinguished (No-EXT) groups. All groups were balanced for trait anxiety using the EPM such that there were no *a priori* significant differences in the time spent on the open arms (*F*_(1,38)_ = 0.03; *p =* 0.86^a^; Fig. 1*B*). Although pre-tone freezing levels for the immediate extinction groups were high, we found this behavior to be common in the literature when extinction training sessions were given shortly after fear conditioning (Maren and Chang, 2006; Chang and Maren, 2009; Chan et al., 2010; Goode et al., 2015). A three-way repeated-measures general linear model (extinction × interval × trial) for the percentage of time spent freezing during exposure to either the extinction training (EXT) or context B (No-Ext) groups found significant, but equivalent changes in freezing behavior, as indicated by a significant main effect of trial (*F*_(2,36)_ = 47.8; *p* < 0.0001^b^) and extinction × trial interaction (*F*_(2,36)_ = 8.47; *p* = 0.001^c^), but no significant interval × trial (*F*_(4,74)_ = 1.53; *p* = 0.21^d^) or extinction × interval × trial (*F*_(4,74)_ = 0.322; *p* = 0.86^e^; Fig. 1*C*) interaction. During the extinction test performed 48 h after conditioning, a two-way ANOVA (extinction × interval) revealed a significant effect of extinction (*F*_(1,42)_ = 18.85, *p* < 0.001^f^), interval (*F*_(2,42)_ = 8.05, *p* = 0.001^g^), and extinction × interval interaction (*F*_(2,55)_ = 3.22, *p* = 0.026^h^; Fig. 1*D*). Fisher’s post hoc tests revealed that extinction applied 30 min after conditioning was ineffective in suppressing CS-elicited fear responses since the level of freezing of animals with extinction was not significantly different than non-extinguished control animals (*p* = 0.63^i^). However, animals were able to extinguish fear responses when extinction training was given after a post-conditioning delay of 3 h (*p* = 0.01^j^) and 24 h (*p* < 0.001^k^). The extinction was more efficient for a delay of 24 h than for 3 h, since animals extinguished 24 h after conditioning exhibited significantly less freezing than animals of the 3-h group (*p* < 0.01^l^) during the test.

Then, to examine the relationship between fear training and extinction, we performed Pearson’s correlational analyses between the percentage of time spent freezing during fear conditioning and during the extinction test. We found a significant positive correlation in animals who received extinction 30 min after conditioning, such that those that exhibited the greatest fear during conditioning also exhibited the greatest deficit (*R* ^2^ = 0.74, *p* = 0.006^m^; Fig. 1*E*). Conversely, in animals who received extinction 24 h after conditioning, we found the opposite correlation, with those who exhibited the least freezing during conditioning exhibiting the highest levels of freezing during the extinction test (*R* ^2^ = 0.52, *p* = 0.044^n^; Fig. 1*F*). Animals that received extinction 3 h after conditioning had no significant correlation between freezing behaviors during the fear conditioning and the extinction test (data not shown; *R* ^2^ = 0.28, *p* = 0.17^o^).

### Intra-BLA infusion of a CRFR_1_ antagonist after conditioning facilitates immediate extinction without interfering with fear learning consolidation

**Figure 2.**
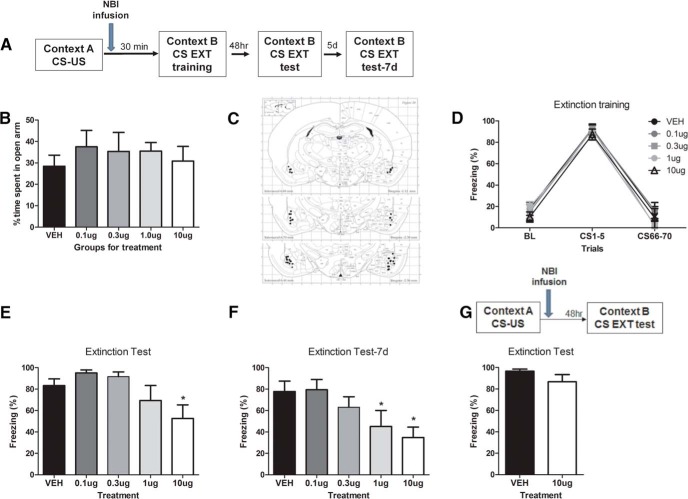
Impaired extinction efficiency due to immediate post-conditioning interval is restored by a post-conditioning bilateral infusion of CRFR_1_ antagonist NBI30775 in the BLA. Animals were separated into groups (***A***) balanced for anxiety-like behavior on the elevated plus maze (***B***). After successful fear conditioning they received a bilateral infusion of NBI30775 or vehicle immediately at the end of the session (***C***) and were given an extinction training session 30 min later (***D***). Animals infused with the 10-µg dose of NBI30775 showed significantly reduced freezing levels during the first testing session 48 h post-conditioning (***E***). Both 1- and 10-µg doses showed significantly reduced freezing levels when tested 1 week later (***F***). A separate group of animals were fear conditioned and received NBI or vehicle afterward and were left undisturbed until testing 48 h later. Treated animals showed similar levels of freezing in the test, suggesting that infusion of NBI does not affect memory consolidation (***G***). BL, baseline freezing during the first 3 min pretone. Data depicted as mean percentage of time spent freezing ± SEM. *Significant difference (*p* < 0.05) with the DMSO vehicle group; *n* = 6–10/group.

Given the above behavioral results, we chose to perform pharmacological experiments (intra-BLA infusion of NBI30775 at doses of 0.1, 0.3, 1, and 10 µg; Fig. 2*A*,*C*) at a postconditioning interval of 30 min with groups balanced for their *a priori* anxiety-like behavior on the EPM (*F*_(4,33)_ = 0.33; *p* = 0.86^p^; Fig. 2*B*). During extinction sessions, all groups significantly decreased their freezing across extinction training trials (*F*_(2,34)_ = 294.8; *p* < 0.0001^q^), with no significant differences in freezing between vehicle- and drug-treated animals (*F* values < 1; Fig. 2*D*). For the extinction test performed 48 h after fear conditioning, a one-way ANOVA revealed a significant effect of the drug infusion (*F*_(4,34)_ = 3.51, *p* = 0.01^r^; Fig. 2*E*). Fisher’s post hoc tests showed that infusion of the highest dose of NBI30775, 10 µg, promoted extinction efficiency, since these animals exhibited significantly less freezing than vehicle-treated control animals (*p* = 0.009^s^; Fig. 2*E*). For the second test performed 1 week after fear conditioning, a one-way ANOVA revealed a significant effect of the prior drug infusion (*F*_(4,34)_ = 3.51, *p* = 0.017^t^). Fisher’s post hoc tests showed that infusion of both 1 µg/0.5 µl and 10 µg/0.5 µl promoted long-term extinction efficiency, since these animals exhibited significantly less freezing than vehicle-treated control animals (*p* = 0.04^u^ and *p* = 0.004^v^, respectively; Fig. 2*F*).

Subsequently, we performed a follow-up experiment to investigate whether the observed effects of the CRFR_1_ antagonist could have been due to interference with the consolidation of fear conditioning. In this experiment, animals were infused with 10 µg of NBI 30775 into the BLA immediately after conditioning and did not receive any extinction session. When they were tested 48 h afterward, NBI-treated animals did not differ in their freezing levels from the vehicle-infused group (Student’s *t*-test *t* = 1.32, *p* = 0.21^w^; Fig. 2*G*).

Taken together, these results reveal that blocking CRF activity in the BLA immediately after fear conditioning facilitates an immediate extinction carried out 30 min after training but does not interfere with normal consolidation processes.

### Intra-BLA infusion of a CRF agonist immediately before a delayed extinction impairs extinction

**Figure 3.**
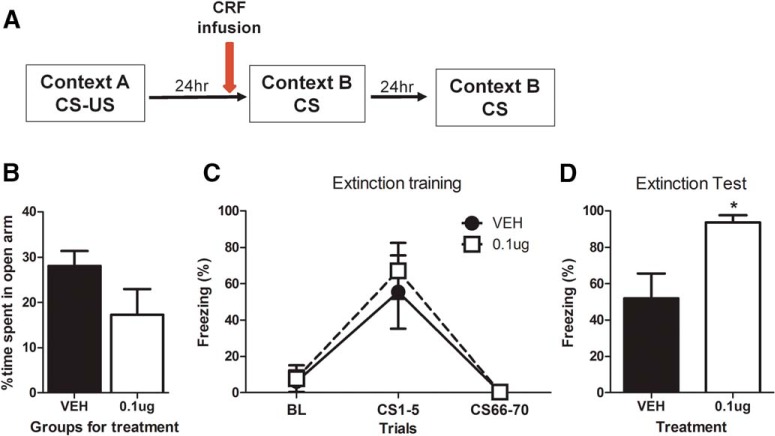
Infusion of CRF agonist CRF_6–33_ in the BLA immediately before delayed extinction session alters extinction efficacy. Animals were separated into groups (***A***) balanced for anxiety-like behavior on the elevated plus maze (***B***), successfully fear conditioned to a similar level (***C***), and infused with CRF_6–33_ or saline 30 min before a delayed (24 h) extinction session. Treated animals showed significantly increased freezing levels compared with vehicle (***D***). BL, baseline freezing during the first 3 min pretone. Data depicted as percentage of time spent freezing ± SEM. *Significant difference (*p* < 0.05) with the CRF agonist CRF_6–33_ group (0.1 µg/0.2 µl); *n* = 5–6/group.

Given the above findings, we then wanted to investigate whether CRH activation was sufficient to produce an extinction deficit. We focused on a delayed extinction protocol (24 h after fear conditioning) in which we found no evidence of an extinction deficit (Fig. 1). We reasoned that if, in immediate extinction protocols, endogenously shock-induced activation of CRH is sufficient for the extinction deficit, then enhancing CRH activation in the BLA before a delayed extinction protocol should also induce a deficit. Thus, we infused a CRF agonist into the BLA 30 min before a delayed extinction session given 24 h after fear conditioning (Fig. 3*A*) in groups balanced for their *a priori* anxiety-like behavior on the EPM (*t* = 1.7; *p* = 0.12^x^; Fig. 3*B*). During extinction training, both groups exhibited significant decreases in freezing behavior, indicated by a main effect of trial (*F*_(2,8)_ = 9.94; *p* = 0.007^y^) at a similar level (interaction and treatment effect *F* values < 1; Fig. 3*C*). As hypothesized, pre-extinction training infusion of 1 µg/0.2 µl of CRF_6–33_ into the BLA altered extinction efficiency, as these animals exhibited significantly more freezing than vehicle-infused animals in the extinction test given 24 h afterward (Student’s *t*-test *t* = 2.90, *p* = 0.028^z^; Fig. 3*D*). These results indicate that infusion of a CRF agonist just before extinction training reduces subsequent fear extinction efficiency.

### Alteration of extinction is correlated with alteration of phosphorylation of the AMPA GluA1 subunits

**Figure 4.**
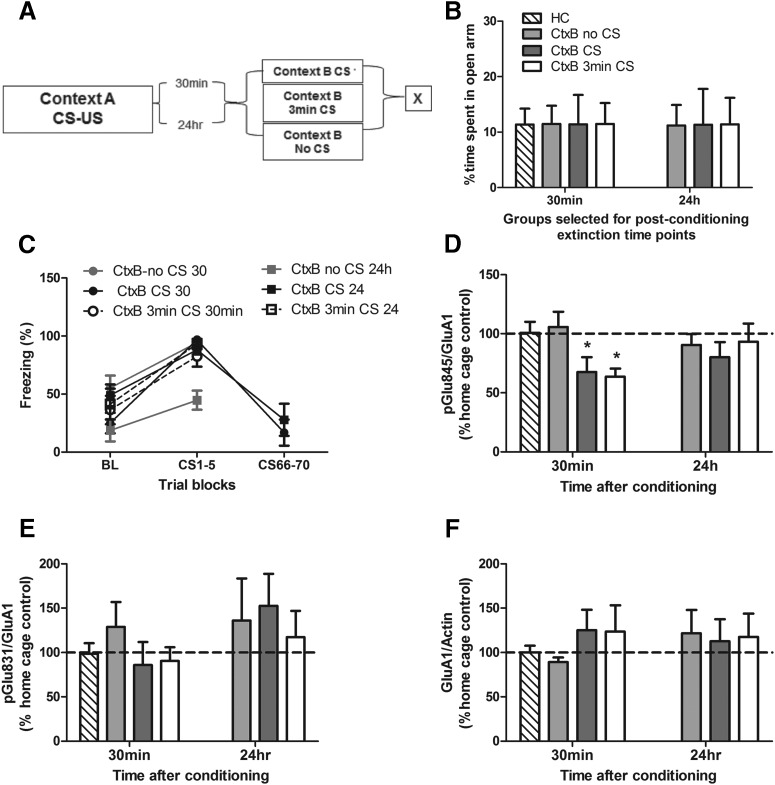
Immediate extinction impairment is associated with reduced phosphorylation of the AMPA GluA1 Ser^845^ subunit. Animals were separated into groups (***A***) balanced for anxiety-like behavior on the elevated plus maze (***B***), fear conditioned and sacrificed (X) immediately after various context B (CtxB) exposures either 30 min or 24 h after training, with each group showing similar levels of freezing during CtxB exposure (***C***). Phosphorylation of AMPA GluA1 Ser^845^ was significantly decreased in animals exposed to CS presentations 30 min post-conditioning but not in those with a delayed (24 h) exposure (***D***) compared with the home cage controls (HC). There were no significant differences in the phosphorylation levels of AMPA GluA1 Ser^831^ (***E***) or total GluA1 receptor protein (***F***). Data depicted as percentage of control group ± SEM. *Significant difference (*p* < 0.05) with HC group; *n* = 6/group.

To uncover possible mechanisms underlying the involvement of the CRF system in the BLA in impaired extinction learning, we examined phosphorylation of AMPA receptors at specific serine residues, as they have been previously linked with extinction learning (Monfils et al., 2009). A new cohort of animals was conditioned and received an extinction session (CtxB CS) either immediately (30 min: CtxB CS 30 min) or after a delay (24 h: CtxB CS 24 h) after the fear conditioning session. Animals were sacrificed at the end of the session (Fig. 4*A*), and Western blots were performed against phosphorylated AMPA receptor subunits. To compare our results with others who have examined phosphorylated AMPA receptor subunit changes after 3 min of CS extinction exposure, we included an additional group (CtxB 3 min CS) in which animals were exposed to 3 min of extinction either 30 min or 24 h after training and took brain samples immediately afterward. To control for possible effects of the new context, an additional group of animals was exposed to Context B without any CS presentations (CtxB no CS). All groups were balanced for their *a priori* anxiety-like behavior on the EPM (*F*_(2,31)_ = .0002; *p* = 0.99^aa^; Fig. 4*B*). During extinction training, all groups exhibited significant decreases in freezing behaviors, indicated by a main effect of trial (*F*_(1,31)_ = 103.1, *p* < 0.0001^bb^), but no significant effects of training interval (*F*_(1,31)_ = 2.18; *p* = 0.15^cc^), group (*F*_(2,31)_ = 2.37; *p* = 0.11^dd^), or interaction (*F*_(2,31)_ = 1.45; *p* = 0.25^ee^; Fig. 4*C*). For the 30-min post-conditioning interval, i.e., when animals exhibited a deficit in fear extinction efficiency, a one-way ANOVA revealed a significant effect of condition (*F*_(3,19)_ = 4.35, *p* = 0.017^ff^). Fisher’s post hoc tests showed that the percentage of phosphorylation of the AMPA GluA1 Ser^845^ subunit in home cage was not significantly different from the CtxtB no CS control group (*p* = 0.75^gg^) but was significantly higher than CtxB CS 30 min and CtxB 3 min CS groups (*p* = 0.042^hh^ and *p* = 0.019^ii^, respectively; Fig. 4*D*), indicating that AMPA GluA1 Ser^845^ phosphorylation is decreased 30 min after fear conditioning. For the 24 h postconditioning interval, i.e., when animals did not show any deficit in fear extinction learning, a one-way ANOVA did not reveal any significant effect (*F* values < 1). Analysis of phosphorylation at another serine residue, GluA1 Ser^831^, found no significant alterations in phosphorylation compared to home cage controls in any condition (*F* values < 1; Fig. 4*E*). Importantly, analysis of the total GluA1 receptors also revealed no significant differences between groups (*F* values < 1; Fig. 4*F*).

### Modulation of AMPA GluA1 Ser^845^ phosphorylation by CRFR_1_ antagonist in the BLA

**Figure 5.**
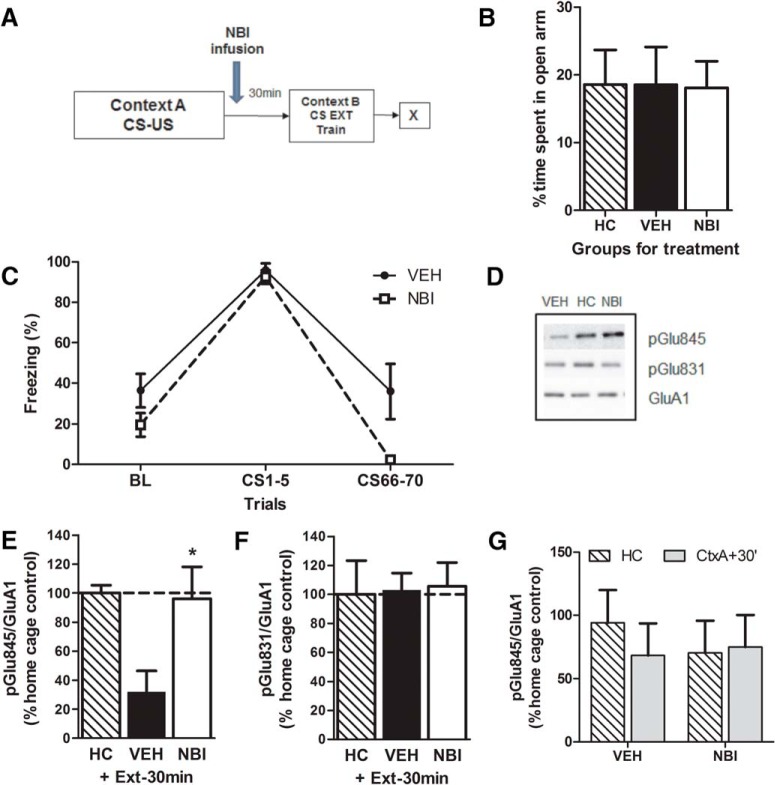
GluA1 Ser^845^ phosphorylation levels are reversed with administration of NBI. Animals were separated into groups (***A***) balanced for anxiety-like behavior on the elevated plus maze (***B***) and received a post-conditioning infusion of either the CRFR_1_ antagonist NBI30775 or vehicle and an immediate extinction session 30 min later (***C***). Treatment with NBI30775 restored GluA1 Ser^845^ phosphorylation to vehicle home cage (HC) levels (***D***,***E***) but had no effect on GluA1 Ser^831^ (***D***,***F***) or on its own (***G***). Data depicted as percentage of vehicle HC control group (hatched bar and also dotted line) ± SEM. *Significant difference (*p* < 0.05) with the vehicle-treated HC group; *n* = 8–12/group.

To determine whether AMPA GluA1 Ser^845^ phosphorylation levels were modulated by the actions of CRF after fear conditioning, we examined phosphorylation in the amygdala in animals infused with either vehicle or NBI30775 and sacrificed after an extinction session delivered 30 min postconditioning (Fig. 5*A*) in groups balanced for their *a priori* anxiety-like behavior on the EPM (*F*_(2,27)_ = 0.004; *p* = 0.99^jj^; Fig. 5*B*). During extinction training, there was a significant main effect of trial (*F*_(2,26)_ = 89.4; *p* < 0.0001^kk^), indicating that both groups decreased their freezing over time. There was also a significant effect of treatment (*F*(1,13) = 7.26; *p* = 0.02^ll^) and a trend for an interaction (*F*_(1.6,20.7)_ = 3.06; *p* = 0.08^mm^; Fig. 5*C*), in which NBI treatment reduced freezing during extinction training. Western blots were performed against phosphorylated AMPA receptor subunits in samples from the amygdala (Fig. 5*D*). Vehicle-treated animals exhibited significantly reduced phosphorylation on the Ser^845^ subunit compared to home cage controls (Student’s *t*-test, *t* = 4.68; *p* < 0.01^nn^; Fig. 5*D*, *E*). Infusion of the CRFR_1_ antagonist NBI30775 restored the phosphorylation levels of this subunit to those of home cage levels (Student’s *t*-test, *t* = 0.18; *p* = 0.86^oo^). Notably, there was no effect of either vehicle or NBI treatment on GluA1 Ser^831^ phosphorylation (Fig. 5*D*, *F*), nor was there an effect of NBI treatment alone on GluA1 Ser^845^ phosphorylation after fear conditioning (*F*_(1, 56)_ = 0.388; *p* = 0.54^pp^; Fig. 5*G*).

### Calcineurin modulates AMPA GluA1 Ser^845^ phosphorylation in the BLA

**Figure 6.**
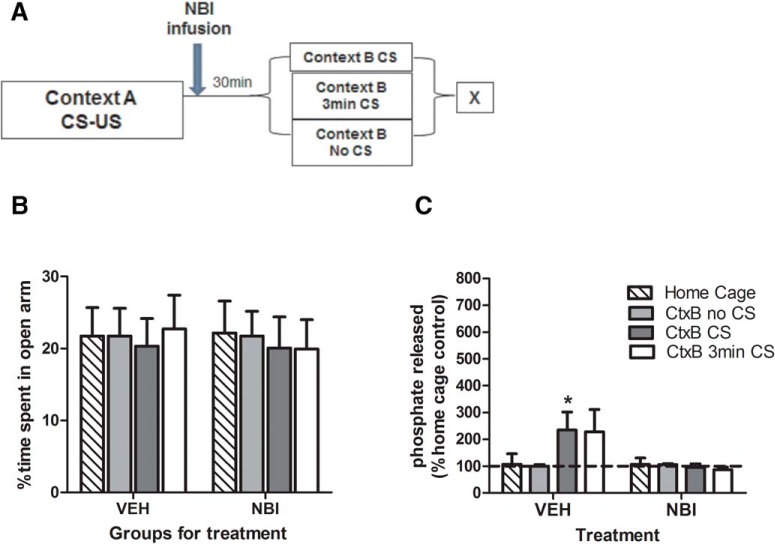
Enhanced calcineurin activity after extinction is reversed by NBI administration. Animals were fear conditioned and infused post-conditioning with either NBI30775 or vehicle, separated into groups (***A***) balanced for anxiety-like behavior on the elevated plus maze (***B***), and sacrificed (X) for calcineurin activity assessment after either 3 min or a full extinction session 30 min after conditioning. Calcineurin activity tended to increase after the extinction session in vehicle-treated animals (CtxB CS group) but was blocked by post-conditioning treatment with NBI30775 (***C***). Data depicted as percentage of vehicle home cage control group (hatched bar and dotted line) ± SEM. *Significant difference (*p* < 0.05) with the vehicle-treated home cage group; *n* = 8–10/group.

We next investigated whether activity of the phosphatase calcineurin might be mediating the actions of CRF on AMPA GluA1 Ser^845^ phosphorylation during extinction. In the 30-min postconditioning interval, as both CtxB 3 min CS and CtxB CS 30 min groups exhibited significantly decreased phosphorylation (see Fig. 4*D*), we examined whether treatment with NBI30775 reduced calcineurin activity, which would allow for the restoration of phosphorylation levels. Animals were divided into groups (Fig. 6*A*) balanced for their *a priori* anxiety-like behavior on the EPM (*F*_(3,69)_ = 0.07; *p* = 0.97^qq^; Fig. 6*B*). Then, they were treated with either NBI30775 or vehicle and sacrificed 30 min postconditioning after either 3 min (CtxB 3 min CS) or a full (CtxB CS 30 min) extinction exposure. A second group of animals received the same behavioral and pharmacological treatments but were sacrificed from the home cage as controls. Treatment with NBI30775 significantly reduced calcineurin activity in the BLA compared with vehicle-treated extinction (CtxB CS 30 min and CtxB 3 min min CS) groups (two-way ANOVA, *F*_(1,67)_ = 6.17; *p* = 0.015^rr^; Fig. 6*C*), indicative of a link between CRF levels and calcineurin activity. Post hoc tests revealed a significant increase in calcineurin activity in the vehicle-treated CtxB CS 30 min group (*p* = 0.02^ss^) that was blocked by NBI treatment (*p* = 0.75^tt^) compared with vehicle-treated home cage controls.

## Discussion

Previous work has highlighted a deficit for extinction programs that are delivered shortly after fear conditioning, as opposed to a better efficiency of delayed extinction sessions (Maren, 2014). Similarly, psychotherapeutic interventions provided soon after a traumatic event have been reported to be rather ineffective in reducing long-term fear responses (Rothbaum and Davis, 2003; Gray and Litz, 2005), although the underlying mechanisms were unclear. Here, we provide strong evidence implicating the CRF system in the BLA as a key mechanism mediating this immediate extinction deficit and identify the phosphorylation of GluA1 and enhanced calcineurin activity as potential downstream mechanisms for CRF actions.

First, in agreement with substantial work in the literature (Maren and Chang, 2006; Woods and Bouton, 2008; Chang and Maren, 2009; Maren 2014), we show here in rats that long-term extinction efficiency is impaired when extinction training occurs shortly (30 min) after fear conditioning, but correctly retained when extinction training is given after a longer delay period (24 h; note that some effectiveness of the extinction training can already be observed at the 3-h time point). Furthermore, individuals exhibiting the greatest amount of freezing during conditioning also show the greatest extinction impairment when the extinction training occurs shortly after fear conditioning, as opposed to the opposite relationship between freezing during conditioning and extinction efficiency when extinction occurs.

In our effort to investigate underlying mechanisms, we reasoned that mechanisms facilitating fear conditioning in the BLA might be at the core of the immediate extinction deficit and postulated that a fear conditioning-activated CRF system in the BLA interferes with the immediate extinction process. This hypothesis was based on previous work implicating CRF in delayed extinction deficits (Gafford et al., 2012) and evidence for a rapid activation of CRF in the amygdala elicited by acute stress, including foot shock (Merali et al., 1998; Yamano et al., 2004), which facilitates fear consolidation processes (Roozendaal et al., 2008; Pitts and Takahashi, 2011; but see Isogawa et al., 2013) through the activation of CRFR_1_ in the BLA (Roozendaal et al., 2002; Hubbard et al., 2007). In agreement with our hypothesis, we found that intra-BLA post-training infusion of the CRFR_1_ antagonist NBI30775 (0.1–10 µg) given shortly after fear conditioning promoted, at the higher doses tested, long-term extinction learning in animals submitted to extinction training 30 min postconditioning and tested for extinction 48 h and 7 d postconditioning. Importantly, the reduced long-term freezing exhibited by animals treated with the CRFR_1_ antagonist is not simply the result of disrupted fear consolidation, as animals just treated with NBI30775 post-conditioning (i.e., not submitted to extinction learning) show freezing levels comparable to vehicle-infused animals when tested 48 h afterward. In supplementary experiments using delayed extinction procedures, we obtained further evidence in support of a causal link between shock-immediacy and/or increased CRF in the BLA and extinction deficits. As with the immediate extinction deficit, we found that the effectiveness of extinction training was impaired when an intra-BLA infusion of the CRF agonist CRF_6–33_ (0.1 µg) was applied just before a delayed (i.e., 24 h postconditioning) extinction training session. Although this evidence supports our line of reasoning, our data do not allow us to exclude the alternative possibility that CRF treatment could have acted as a CS on the extinction session, inducing some sort of aversive conditioning to context B, which would then be manifested as increased freezing during the extinction testing session. Further experiments including extinction testing in a different “C” context are warranted for unambiguously concluding the impact of increased CRF on extinction efficiency.

Therefore, we identify here the activation of CRFR_1_ in the BLA as a key mechanism interfering with the effectiveness of immediate fear extinction training. This novel finding fits with previous reports that have implicated the BLA CRF system in impaired extinction in delayed extinction training paradigms (Gafford et al., 2012; Abiri et al., 2014) and fits with the view that the degree of fear individuals experience just before the onset of an extinction session might determine the efficacy of extinction learning (Maren and Chang, 2006; Maren, 2014). Although whether BLA CRFR_1_ activation precisely reflects the CS-related degree of amygdalar excitation remains to be established, intra-BLA CRF infusions were shown to lead to robust increases in anxiety behaviors (Sajdyk et al., 1999) and to induce long-lasting sensitization of noradrenergic substrates and PTSD-like symptoms (Rajbhandari et al., 2015) in rodents. Moreover, in vivo release of CRF in the BLA has been found to correlate with the level of freezing behavior in response to fear conditioning experiments (Mountney et al., 2011).

In addition, we show evidence implicating a decrease in phosphorylation of the GluA1 glutamate receptors at Ser^845^, but not Ser^831^, as a downstream mechanism of BLA CRFR_1_ implication in the immediate fear extinction deficit. GluA1 membrane insertion was shown to be required for fear conditioning–induced synaptic plasticity and consolidation (Rumpel et al., 2005) and regulated by GluA1 phosphorylation at Ser^845^ (Blackstone et al., 1994). Importantly, a transient up-regulation of GluA1 phosphorylation at Ser^845^ has been critically implicated in the susceptibility of long-term expressed memories to fear erasure by manipulations involving reconsolidation (Clem and Huganir, 2010) or extinction (Monfils et al., 2009) protocols after a brief retrieval of the fear memory. Conversely, administration of two retrieval sessions of a long-term established auditory fear memory close in time (i.e., the second within 1 h after the first retrieval session) led to a rapid dephosphorylation of GluA1 at Ser^845^ that was associated with the inability to induce memory-impairing effects (i.e., fear memory reconsolidation) by a protein synthesis inhibition (Jarome et al., 2012). These data suggest that the second retrieval rapidly altered the phosphorylation state of GluA1. This fits with our findings that, whereas fear-conditioned animals placed in a novel context 30 min post-training showed similar levels as home cage controls, those exposed to either a short (3-min) or long (30-min) extinction protocol displayed dephosphorylation of GluA1 at Ser^845^ in the BLA. Importantly, the same extinction treatments did not affect the phosphorylation rate when they were given 24 h after fear conditioning, further supporting a potential role of dephosphorylation of GluA1 at Ser^845^ in the BLA in the immediate extinction deficit. As observed in our study, the proximity of the CS application in the short and long extinction sessions to fear conditioning in the immediate fear extinction deficit phenomenon mimics mechanisms underlying the repetition of stimuli and conditions described by Jarome et al. (2012) that make the fear memory resistant to erasure. The rapid dephosphorylation of GluA1, as observed in our study, has been linked to α-amino-3-hydroxy-5-methyl-4-isoxazolepropionic acid (AMPA) receptor endocytosis, leading to alterations in synaptic strength (Ehlers 2000) and shown to depend on increased activity of the protein phosphatase 2B or calcineurin (Ehlers 2000; Snyder et al. 2003). In full agreement with these findings, we observed increased calcineurin activity in the BLA in the groups submitted to short (3-min) or long (30-min) extinction protocols starting 30 min after fear conditioning, the same time points that also display a dephosphorylation of GluR_1_ at Ser845. Calcineurin has been previously linked to the regulation of anxiety and fear conditioning in the amygdala (Lin et al., 2003; Baumgärtel et al., 2008). Importantly, intra-BLA infusion of NBI30775 immediately after fear training (at the dose of 10 µg that enables efficient extinction in the immediate extinction protocol) in animals that were exposed to extinction training 30 min after fear conditioning prevented (1) the dephosphorylation GluA1 at Ser^845^ and (2) the increase in calcineurin activity in the BLA observed in vehicle-treated animals after extinction. Therefore, our findings suggest a possible mechanism whereby fear conditioning–induced enhancement of CRF and activation of CRFR_1_ in the BLA may act to prevent immediate extinction learning by blocking GluA1 insertion into the synapse via targeted dephosphorylated GluA1 AMPA subunits by enhanced calcineurin activity.

Although CRFR_1_ is primarily associated with increased production of cyclic AMP (cAMP) through adenyl cyclases, studies have shown that it can also interact and influence other G-protein systems, such as those of protein phosphokinases, modifying the balance between several signaling cascades rather than just one pathway, in a tissue-specific manner (Grammatopoulos and Chrousos, 2002; Gallagher et al., 2008). Evidence from the literature points to spiny pyramidal glutamatergic projection neurons in the BLA as particularly involved in the CRFR_1_-mediated effects reported in this study. For example, administration of CRF into the BLA produced a specific dose-dependent increase in the expression of cFos-ir in pyramidal neurons (Rostkowski et al., 2013). In addition, calcineurin is predominantly found in pyramidal neurons in the BLA (Leitermann et al., 2012). The involvement of BLA projection neurons is particularly relevant in the context of extinction learning, as substantial work shows that the BLA regulates the consolidation of fear extinction not only through local mechanisms, but also through reciprocal projections to other brain regions, particularly the medial prefrontal cortex (Akirav and Maroun, 2007; Quirk and Mueller, 2008; Herry et al., 2010; Pape and Pare, 2010). In fact, the medial prefrontal cortex has been critically implicated in the encoding and retrieval of extinction (Maren, 2014) and with stress-induced morphological changes associated with impaired extinction (Izquierdo et al., 2006; Miracle et al., 2006; Wilber et al., 2011). Furthermore, studies have identified links between BLA activity and medial prefrontal cortex function (Dilgen et al., 2013). Thus, in the case of immediate extinction deficit, it has been proposed that amygdalar hyperexcitability may inhibit medial prefrontal cortex circuitry and interfere with extinction retrieval. Given the ability of CRF to render the BLA excitable for long periods of time (Rainnie et al., 1992; Sandi et al., 2008), our data support this hypothesis via a CRF-mediated mechanism.

Immediate extinction therapies have been offered as a potential solution to combat the development of PTSD in individuals exposed to traumatic events. Studies from animal research have demonstrated that these kinds of therapies may not be effective. In this study, we go beyond the behavioral level and identify the activation of CRFR_1_ in the BLA as a critical mechanism underlying this phenomenon. Our findings highlight the treatment with CRFR_1_ antagonists as a potential adjuvant capable to improve the effectiveness of behavioral therapies given shortly after exposure to trauma.

## Synthesis

The decision was a result of the Reviewing Editor Tallie Z. Baram and the peer reviewers coming together and discussing their recommendations until a consensus was reached. A fact-based synthesis statement explaining their decision and outlining what is needed to prepare a revision is listed below. The following reviewers agreed to reveal their identity: Victoria Risbrough, Ki Goosens

Both reviewers, as well as myself, appreciated the substantive revisions to the paper.One of the reviewers made a few suggestions to further improve the manuscript. Please consider them.

1) The statistics showing no group effect for each set of EPM data need to be reported either in the text or Table 1.

2) I am still not convinced that the data in Figure 3 are relevant to the core hypotheses being addressed about immediate extinction. The author's rebuttal letter states, "The rationale for this experiment is the one indicated in the manuscript", yet I don't see a rationale for this experiment in the paper. The only text I see states, "we aimed to investigate whether increasing CRF actions in the BLA during delayed extinction learning would affect extinction". This is a rationale that states that this experiment explains *delayed* extinction; it does not say anything about immediate extinction. Unless the authors can present, in the manuscript text, a compelling rationale for including this experiment, I believe it should be omitted.

3) line 307 "delayed..." should read " at a delay..."

3) Figures 2D and 3C should have legends to clarify which symbols correspond to each group.

4) Figure 4C "homecage" should read "home cage"

5) There are many places where the authors use the term "training" and it is not clear whether it refer to fear conditioning or extinction training. Please use a more specific term than "training" throughout the text.

6) The text in the manuscript should specify that the high pre-tone freezing levels during immediate extinction are similar to what others have reported and provide citations.
